# Nondaily smoking: a population-based, longitudinal study of stability and predictors

**DOI:** 10.1186/1471-2458-14-123

**Published:** 2014-02-05

**Authors:** Elisabeth Kvaavik, Tilmann von Soest, Willy Pedersen

**Affiliations:** 1 Norwegian Institute for Alcohol and Drug Research, P.O. Box 565, Sentrum N-0105, Oslo, Norway; 2 Department of Psychology, University of Oslo, P.O. Box 1094, Blindern N-0317, Oslo, Norway; 3 Department of Sociology and Human Geography, University of Oslo, P.O. Box 1096, Blindern N-0317, Oslo, Norway

**Keywords:** Nondaily smoking, Adolescence, Longitudinal, Stability

## Abstract

**Background:**

Nondaily smoking appears to have remained stable in Western countries in recent years, alongside a steep decline in daily smoking. Nondaily smoking increases the risk of several diseases and premature mortality, but our knowledge about nondaily smoking is limited. The present study was designed to examine the stability of nondaily smoking during young adulthood, and to identify adolescent factors predictive of nondaily smoking compared with nonsmoking and non-nicotine-dependent and nicotine-dependent daily smoking.

**Methods:**

A population-based sample (n = 942) of Norwegians was followed up by surveys for 13 years, from adolescence to young adulthood. Information about smoking patterns, nicotine dependence, school achievement, parents’ and peers’ smoking, and parental monitoring was collected. Data on parental and participants’ education were obtained from a national register.

**Results:**

Of all nondaily smokers at age 21 years, 26% were still nondaily smokers at 27 years, while 17% had become daily smokers and 57% had quit. Bivariate analyses revealed that young adult nondaily smokers did not differ from nonsmokers on any of the included variables, while a number of differences in parental, peers’ and individual characteristics were observed between nondaily smokers and the two categories of smokers in young adulthood. Longitudinal analyses revealed that unorganized leisure time activities and peers’ smoking differentiated nondaily smoking from nonsmoking. Higher educational achievement and less parental binge drinking predicted nondaily smoking and differentiated it from both categories of daily smoking.

**Conclusions:**

The degree of nondaily smoking-stability from 21 to 27 years of age was modest, and most nondaily smokers quit smoking in the course of young adulthood. Young adult nondaily smokers were quite similar to nonsmokers, but differed substantially from both nicotine-dependent and nondependent daily smokers. The study suggests that nondaily smoking—at least in the absence of traditional risk factors for smoking—is usually a transitory behavior, with most people returning to nonsmoking.

## Background

Nondaily smoking is less prevalent than daily smoking [1,2], but appears to have remained stable or perhaps increased in some Western countries in recent years, while daily smoking has steeply declined [3,4]. Nondaily smoking has been shown to increase the risk of several diseases and mortality, although to a lesser degree than daily smoking [5], and is also reported to be predictive of daily smoking [2]. However, our knowledge about nondaily smoking is limited; few studies have examined the long-term stability of nondaily smoking [6-9], and we lack longitudinal studies that predict young adult nondaily smoking based on teenage characteristics [10,11], and studies from countries other than the US. The present study draws on population-based Norwegian data, and follows a sample from their mid-teens until their late twenties.

In the literature, nondaily smokers are given varying definitions and labels. Typically, they are named social, occasional, intermittent, very low rate, casual or recreational smokers [2,3,12], illustrating the different views of this group. In addition, nondaily smokers often consider themselves to be nonsmokers, and therefore identifying such smokers by self-report can be challenging [3,13,14]. In the current study, “nondaily smokers” are those who reported that they smoke, but not daily. Thus, the definition excludes low-rate daily smoking.

Until recently, cigarette smoking was considered primarily using the model of dependence. Repeated use of a substance might lead to adaptation, tolerance and dependence, and subsequent withdrawal symptoms if blood levels drop. This encourages the person to maintain the supply of nicotine by frequently smoking cigarettes [4]. Nondaily smoking, however, is not well explained by such models. The drop in blood nicotine levels would be expected to result in withdrawal symptoms and motivate more frequent smoking, yet nondaily smokers report low nicotine dependence compared with daily smokers [15,16]. Thus, nicotine dependence does not seem to be a sufficient cause for continued nondaily smoking. As a result, nondaily smoking has often been conceptualized either as a transitional stage to daily smoking or as a step in a gradual reduction of daily smoking [4].

Longitudinal studies indicate nondaily smoking to be more stable than would be expected according to the dependence model. For instance, one US study showed that 40% of adult occasional smokers maintained their habit over a two-year span [7], and another found that about two-thirds of all occasional smokers had also smoked occasionally two years earlier [6]. A one-year follow-up study from Sweden found that 60% of those who had been intermittent smokers at baseline were still intermittent smokers at follow-up [9], while a Norwegian study following adolescents for seven years from the age of 13 years, concluded that about 40% of those who were recreational smokers at some time in adolescence, continued to be so at the age of 20 years [8]. In conclusion, several studies indicate that nondaily smoking might have moderate to high stability, at least over a couple of years. Studies that have followed nondaily smokers for longer periods are scarce, and therefore required.

Another important issue is how nondaily smokers differ from nonsmokers and daily smokers. Cross-sectional findings, mostly from the US, have shown that nondaily smokers are quite similar to nonsmokers, with the exception that they may have a less favorable mental health profile [17]. Compared with daily smokers, nondaily smokers are younger, more often females, more highly educated, and have higher incomes [3,18,19]. In addition, they less frequently consider themselves to be “smokers” [15,16,18-20]. However, the cross-sectional nature of these studies precludes drawing conclusions about causality.

The few longitudinal studies conducted have mainly examined the importance of factors such as school achievement, conduct problems and alcohol and drug use in adolescence for the development of nondaily smoking. The results indicate that a low level of these problems predicts nondaily smoking when compared with daily smoking, but that the predictive capacity for nondaily smoking versus nonsmoking is small [8,10,11]. Other longitudinal studies comparing subsequent nondaily and daily smoking have reported that this pattern is also predicted by lower levels of parental and peer smoking [8,11], higher educational aspirations [10] and more cultural and social resources [8]. With the exception of peer smoking, these factors do not discriminate between nondaily smoking and nonsmoking [8,11].

Little information is available to determine whether nondaily smokers differ from all or only some groups of daily smokers. To date, studies have indicated that light daily smokers (1–9 cigarettes per day) have lower socioeconomic status (SES) than nondaily smokers, and moderate to heavy daily smokers (>9 cigarettes per day) have even lower SES [2]. Only a few longitudinal studies that compare nondaily smokers with different groups of daily smokers have been conducted. In the present study, we compared nondaily smokers with smokers with different degrees of nicotine dependence.

The aims of the present study were as follows.

1. To examine the stability of nondaily smoking from 21 to 27 years.

2. To identify correlates and longitudinal predictors of nondaily smoking, compared with both nonsmoking and daily smoking.

3. To examine whether nondaily smokers differ from daily smokers in general, or whether nondaily smokers in particular differ from nicotine-dependent smokers.

## Methods

### Procedure and participants

This research is based on the *Young in Norway Longitudinal Study*, which has been described elsewhere [21]. In short, a population-based sample of Norwegian adolescents was followed over 13 years with four data collections, from mid-adolescence in 1992 until their late twenties in 2005. To obtain information about participants’ and parental education, the data set was linked with Statistics Norway’s Historical Event Database. The study was approved by the Regional Ethics Committee for Health Research and the Norwegian Data Inspectorate, and all participants gave their written consent for participation.

The initial response rate was 97%, and the cumulative response rate over all four data collections was 69%. When the attrition from the Statistics Norway’s Historical Event Database was taken into consideration, the overall response was 60%. In the analyses, we draw upon data from 942 persons who were initially in 7th or 8th grade in junior high school; 410 males (43.5%) and 532 females (56.5%). We used data from three waves; 1994 (T1, mean age 15.0 years (*SD* = 0.6)), 1999 (T2, mean age 20.7 years), and 2005 (T3, mean age 26.7 years).

### Instruments

#### Dependent and independent variables

##### Smoking and nicotine dependence

At all data collections, smoking was assessed by the question *Do you smoke?* Response alternatives were: (1) *I have never smoked*, (2) *I have never smoked regularly and do not smoke now*, (3) *I have smoked regularly, but have quit now*, (4) *I smoke, but not daily* and (5) *I smoke daily, about......cigarettes*. Categories 1, 2 and 3 were labeled “nonsmokers”. Category 4 was labeled “nondaily smokers”. Smoking status at T1 was used as an independent variable when aiming at predicting nondaily smoking at T2, while smoking status at T2 was used as an independent variable when aiming at predicting nondaily smoking at T3. Nicotine dependence was assessed at T2 and T3 using the Fagerström Test for Nicotine Dependence, with the cutoff set at ≥ 4 [22]. This allowed us to distinguish between those who were not nicotine dependent, and those who were nicotine-dependent daily smokers.

#### Independent variables

##### School grades

At T1, school grades in mathematics, and Norwegian and English languages were assessed by self-report.

##### Education

Parental educational level when the participants were 16 years old (about T1) was assessed by register data and coded using four categories describing the highest level of education the father and/or mother had completed: (1) elementary school, (2) high school, (3) lower grade at college/university, and (4) higher grade at college/university. Participants’ education at T2 was coded into five levels according to the highest level of education completed: (1) elementary/secondary school (9 years), (2) high school (12 years), (3) high school, comprehensive/upper (13 years), (4) college/university, lower grade (14–17 years), and (5) college/university, higher grade (>17 years). Dropping out of high school was defined as not having completed high school before 21 years of age.

##### Parental and best friends’ smoking

The participants were at T2 asked whether their father and mother had smoked during the participant’s childhood*,* with four response categories; *“No, never”, “One cigarette, cigar or pipe rarely”, “Has smoked regularly, but has quit now”,* and *“Yes, daily”*. The first three response categories were combined and mothers’ and fathers’ smoking were combined. Parental smoking was then categorized as “both parents are never, former or occasional smokers” or “one or both parents are daily smokers”. Best friends’ smoking at T1, T2 and T3 was categorized as “having at least one best friend/boyfriend/girlfriend who smoked regularly” or “having no best friend/boyfriend/girlfriend who smoked”.

##### Parental alcohol intoxication

Parental alcohol intoxication was measured at all three data collections by the question “*Have you ever seen your parents drunk*?” with five response alternatives; *“Never”, “A few times”, “A few times a year”, “A few times a month”* and *“A few times a week”.* The values were summed over the three data collections and used as a continuous variable (range = 0–12).

##### Parental monitoring

An instrument to measure parental monitoring contained six statements about perceived parental norms and parental knowledge of the adolescent’s actions at T1. The statements are “*My parents usually know where I am and what I do in the weekends*”, “*My parents pretty much knows who I spend my spare time with*”, “*My parents know most of the friends I’m with on my spare time*”, “*My parents usually know where I am and what I do on the weekdays*”, “*My parents like most of the friends I’m with on my spare time*” and “*It’s important to my parents that they know where I am and what I do in my spare time*”. The six response alternatives ranged from 0 (*Not right at all*) to 5 (*Totally right*) and were summed to obtain a parental control score (range = 0–30).

##### Leisure time

Unorganized leisure time at T1 was measured by four questions about the frequency (times per week) the following activities; “*spent time at café or snack bars*”, “*spent most of the evenings out with friends*”, “*been driving (yourself) or getting a ride with someone with a car, motorbike, or moped just for fun*” and “*going to the city center/downtown*”. The answers were summed to an index with values ranging from 0 to 28.

### Statistical analyses

Descriptive statistics were used to examine the degree of stability of nondaily smoking from T2 to T3. (At T1, it was assumed that smoking habits may not yet have been established, and we decided not to study the stability from T1 to T2.) Chi-square analyses were used to assess associations between smoking categories and categorical variables at T2 and T3. Analyses of variance (ANOVA) were conducted to examine differences between the four smoking categories at T2 and T3 for continuous variables. Bonferroni post hoc tests were performed to examine differences between groups. As a next step, we conducted multinomial regression analyses, where smoking status at T2 and T3 were dependent variables. Family factors, peers’ smoking and a number of individual characteristics measured at T1 and T2 were predictor variables. Nondaily smokers were first contrasted with nonsmokers, then with nondependent and finally to nicotine-dependent daily smokers. For each regression analysis, two models were estimated; the first without and the second with control for previous smoking status. All models were adjusted for gender and age. The predictors were removed one by one by backward deletion until only significant variables remained. We also tested whether the associations between predictor variables and outcome variables differed for men and women using logistic regression analyses. Because there was no evidence of interactions for any predictor variable (p > 0.05), males and females were combined in the analyses. Previous smoking status was controlled for in the last model to avoid possible confounding and a potential effect of high stability of smoking.

## Results

At T2 (mean age 21 years), 59% of participants were nonsmokers, 13% were nondaily smokers, 17% were nondependent daily smokers and 11% were nicotine-dependent daily smokers. Among men, 60% were nonsmokers, 15% were nondaily smokers and 25% were daily smokers at T2. Corresponding numbers for women were 58%, 12% and 30%, respectively. At T3 (mean age 27 years), 10% of both genders were nondaily smokers. Transitions between the smoking categories from T2 to T3 are presented in Table 1. The stability of nondaily smoking during young adulthood was low, with 26% of T2 nondaily smokers being nondaily smokers at T3, and more than half of them quitting. In contrast, 90% of nonsmokers at T2 were still nonsmokers at T3.

**Table 1:** Smoking patterns of men and women at T3 by smoking patterns at T2 (n = 942)

	**T3 (mean age 26.7 years)**			
** T2 (mean age 20.7 years)**	**Nonsmokers**	**Nondaily smokers**	**Daily smokers NND**	**Daily smokers ND**
	**(n = 636)**	**(n = 93**)	**(n = 120)**	**(n = 93)**
	**n (%)**	**n (%)**	**n (%)**	**n (%)**
**Nonsmokers (n = 553)**	498 (90.1)	26 (4.7)	17 (3.1)	12 (2.2)
**Nondaily smokers (n = 125)**	71 (56.8)	33 (26.4)	18 (14.4)	3 (2.4)
**Daily smokers NND (n = 156)**	51 (32.7)	24 (15.4)	58 (37.2)	23 (14.7)
**Daily smokers ND (n = 108)**	16 (14.8)	10 (9.3)	27 (25.0)	55 (50.9)

*NND* not nicotine dependent, *ND* nicotine dependent.

Table 2 shows the predictor variables for all four smoking groups at T2 (corresponding results for T3, not shown, were similar). The table reveals overall group differences for all variables included in the analyses. Post hoc tests showed that nondaily smokers in young adulthood did not differ from nonsmokers on any variable. However, they differed from nondependent daily smokers by education (p = 0.001), school grades (p = 0.001) and unorganized leisure time (p < 0.001) with nondaily smokers having the more favorable outcomes. Further, nondaily smokers differed from nicotine-dependent smokers by all these variables and on parental education (p = 0.004), parental alcohol intoxication (p < 0.001) and parental monitoring (p = 0.011), also with nondaily smokers having the more favorable outcomes for all variables.

**Table 2:** Characteristics of 942 nondaily smokers, nonsmokers and daily smokers at age 21 years (T2)

	**Nondaily smokers (n = 125)**	**Nonsmokers (n = 553)**	**Daily smokers NND (n = 156)**	**Daily smokers ND (n = 108)**	**p-value for test of differences between groups†**
**Family factors at T1**					
Gender, % women	52.0	55.5	68.6	49.1	0.005
Parental educational level, M (SD)	2.6 (0.9)	2.5 (0.8)	2.4 (0.8)	2.2 (0.7)**	0.007
Parental alcohol intoxication, M (SD)	2.1 (2.1)	2.0 (2.1)	2.6 (2.2)	3.2 (2.3)***	<0.001
Parental control, M (SD)	23.4 (5.3)	24.2 (4.8)	21.8 (6.1)	21.2 (5.8)*	<0.001
One or both parents nondaily or daily smokers, %	62.4	60.8	75.6	76.6	<0.001
**Peers smoking status at T2**					
One or two best friends smokers, %	57.6	42.3	87.2	96.3	<0.001
**Individual characteristics**					
Educational level at T2, M (SD)	3.9 (0.5)	3.9 (0.5)	3.6 (0.6)**	3.6 (0.6)***	<0.001
High school dropout at T2, %	18.6	16.0	32.2	40.4	<0.001
School grades at T1, M (SD)	3.5 (0.5)	3.5 (0.5)	3.2 (0.5)**	3.1 (0.6)***	<0.001
Unorganized leisure time T1, M (SD)	5.0 (4.0)	4.1 (3.7)	7.4 (5.4)***	8.6 (5.8)***	<0.001
T1 smoking status, %					
Nonsmokers	69.6	91.5	50.6	48.1	<0.001
Nondaily smokers	24.8	5.4	23.7	14.8	
Daily smokers	5.6	3.1	25.6	37.0	

†Results show ANOVA for continuous variables, and chi-square analyses for categorical variables. Asterisks denote significant difference from nondaily smokers: *p < 0.05, **p < 0.01, ***p < 0.001. *NND* not nicotine dependent, *ND* nicotine dependent.

Table 3 shows that high levels of unorganized leisure time activities in adolescence at T1 (mean age 15 years) predicted nondaily smoking rather than nonsmoking in young adulthood, at T2 and T3 (not adjusted for earlier smoking status), using multinomial regression analyses. Peer smoking was associated with a higher probability of subsequent nondaily smoking, compared with nonsmoking, at T3, but not at T2. Nondaily smokers at T2 and T3 differed from nondependent daily smokers in having less unorganized leisure time and higher educational levels, whereas higher school grades only predicted nondaily smoking at T2, not at T3. Finally, when nondaily smoking was compared with nicotine-dependent smoking, we found that nondaily smoking at T2 was predicted by higher school grades, lower levels of parental alcohol intoxication, and lower levels of unorganized leisure activities. Nondaily smoking at T3 was predicted by higher education and less peer smoking.

**Table 3:** Predictive values of social and behavioral factors on nondaily smoking at T2 and T3 (n = 942)

	**Nondaily smoking versus nonsmoking**		**Nondaily smoking versus daily smoking without nicotine dependence**		**Nondaily smoking versus daily smoking with nicotine dependence**	
**T2 (mean age 20.7 years)**						
*Predictors at previous time points*	*Multiple model*	*Multiple model adjusted for T1 smoking*	*Multiple model*	*Multiple model adjusted for T1 smoking*	*Multiple model*	*Multiple model adjusted for T1 smoking*
School grades (T1)			1.69 (1.04–2.75)		2.57 (1.54–4.28)	2.15 (1.26–3.66)
Participants’ education (T2)			1.90 (1.13–3.19)	1.87 (1.11–3.17)		
Parental alcohol intoxication (T1)					0.83 (0.73–0.94)	0.83 (0.73–0.94)
Unorganized leisure time (T1)	1.06 (1.01–1.12)		0.91 (0.86–0.97)	0.93 (0.88–0.99)	0.90 (0.85–0.95)	0.91 (0.85–0.97)
*Smoking status (T1)*	–		–		–	
Nonsmokers	–	Reference group	–	Reference group	–	Reference group
Nondaily smokers	–	6.50 (3.53–11.98)	–		–	
Daily smokers	–		–	0.22 (0.08–0.60)	–	0.25 (0.09–0.68)
**T3 (mean age 26.7 years)**						
*Predictors at previous time points*	*Multiple model*	*Multiple model adjusted for T2 smoking*	*Multiple model*	*Multiple model adjusted for T2 smoking*	*Multiple model*	*Multiple model adjusted for T2 smoking*
Participants’ education (T2)			1.91 (1.13–3.21)		2.19 (1.27–3.77)	
Smoking friends (T2)	2.04 (1.27–3.27)				0.25 (0.11–0.58)	
Unorganized leisure time (T1)	1.09 (1.03–1.15)	1.06 (1.00–1.12)	0.93 (0.88–0.99)			
*Smoking status (T2)*	–		–		–	
Nonsmokers	–	Reference group	–	Reference group	–	Reference group
Nondaily smokers	–	8.41 (4.72–14.97)	–		–	5.11 (1.29–20.20)
DS FTND < 4	–	7.26 (3.73–14.16)	–	0.33 (0.15–0.73)	–	
DS FTND ≥ 4	–	9.74 (3.83–24.77)	–	0.30 (0.11–0.79)	–	0.11 (0.04–0.31)

All values are odds ratios (95% confidence interval) resulted from multiple multinomial regression analyses. Models include only variables significantly predicting nondaily smoking, adjusted for age and gender and mutually for all included variables. Excluded variables at T2: parental education, high school dropout, parental smoking, smoking friends and parental control. Excluded variables at T3: parental education, school grades, parental smoking, parental drunkenness, and parental control.

After inclusion of initial smoking status in the models at T2, unorganized leisure time no longer differentiated between nondaily smoking and nonsmoking in young adulthood. School grades lost their significance in the prediction of nondaily smoking versus daily smoking without nicotine dependence. At T3, all predictors became non-significant when earlier smoking was controlled, except unorganized leisure time, which still differentiated between nondaily smoking and nonsmoking.

## Discussion

First, we found that the stability of nondaily smoking over a six-year period in young adulthood was modest: One in four remained nondaily smokers, and the majority quit smoking, while 15% became daily smokers. Second, when young adult nondaily smokers were compared with nonsmokers in cross-sectional analysis, we did not find any differences in any of the included variables. Young adult nondaily smokers did, however, differ from daily smokers with regard to several family and individual characteristics, such as parental education, school grades and school dropout rates. The differences between nondaily smokers and nondependent daily smokers in young adulthood were less pronounced than the differences between nondaily smokers and nicotine-dependent smokers. Third, longitudinal analyses indicated that only unorganized leisure time activities and peers’ smoking in adolescence predicted nondaily smoking compared with nonsmoking in young adulthood, while a number of family, peer and individual characteristics predicted nondaily smoking compared with daily smoking.

The study suggests that nondaily smoking assessed in young adulthood often is a transitory behavior. Nondaily smoking often seems to be a response to peer influence and unorganized leisure time activities, with little social control. Young adults who take part in nondaily smoking are quite similar to nonsmokers; they do, however, differ markedly from daily smokers. Thus, the processes and risk factors behind the development of nondaily smoking seem to be different from those leading to daily smoking. In the absence of more established risk factors for smoking, nondaily smoking in young adulthood will often be a passing experience, and nondaily smokers without such risk factors will often quit smoking.

### Modest stability of nondaily smoking

The stability of nondaily smoking during young adulthood in our study was lower than in previous studies. However, the follow-up period in earlier studies was shorter, which might, in part, explain the different results [6,7,9]. A previous Norwegian longitudinal study with a longer follow-up period also found higher stability than we did [8]. This may be because our study was conducted at a time of decreasing smoking prevalence in Norway [23], whereas the previous study followed the participants in the 1980s and 1990s, when no such decrease was observed [23]. Thus, the lack of stability of nondaily smoking in our study may reflect two different processes: a general tendency to go in and out of this smoking status, and a possible period-specific tendency to quit all forms of smoking. In addition, the young adult nondaily smokers in the current study might also be late initiators of daily smoking.

We found that most young adult nondaily smokers quit, which may indicate that nicotine dependence plays a moderate role in their future smoking behavior. On the other hand, 17% of nondaily smokers became daily smokers, indicating that their actual control over their smoking may be lower than imagined [16,24]. Contemporary explanations of addiction often posit that addicted persons are motivated to continue taking drugs to experience the positive effects of the drug or to avoid withdrawal symptoms [25]. However, in their theory of addiction, Robinson and Berridge suggest that the process is more complex [26]. They argue that potentially addictive drugs share the ability to alter brain organization, and the critical neuroadaptations for addiction render important brain rewards systems “sensitized” to drugs and to drug-associated stimuli. Recent genetic studies suggest that nicotine receptors and genes also play an important role in addiction [27]. Thus, nondaily smoking may rapidly lead to such “sensitizing” in genetically vulnerable subjects. However, the finding that most young adult nondaily smokers in the current study quit smoking indicates that the majority of nondaily smokers do not develop nicotine dependence, and that nondaily smoking is not typically a stage leading to daily smoking.

### Characteristics of nondaily smokers

Consistent with previous research, we found few differences between nonsmokers and nondaily smokers in young adulthood, while nondaily smokers differed from daily smokers with regard to several characteristics, such as parental smoking, and their own school achievement and education, with nondaily smokers having the more favorable outcomes [12,18,19]. We also found that women were underrepresented among young adult nondaily smokers. This finding is in contrast to previous studies, which have found females to be overrepresented among nondaily smokers [3,12,17,19]. However, in Sweden, a pattern similar to our findings has been observed [28]. An explanation might be the high prevalence of snus use among men in Norway and Sweden [29,30], which may be a substitute for both occasional and daily smoking. One should also note that the snus users represent a more resourceful group (by having higher social status and/or higher education) than the daily smokers [31]. Further, a previous study from Norway reported that a combination of daily snus use and nondaily smoking among men was common [26]. Thus, one may witness a new pattern of combined snus use and nondaily smoking in more resourceful groups than those who are currently daily cigarette smokers.

In the current study, only unorganized leisure time activities and peers’ smoking in adolescence differentiated between nondaily smoking and nonsmoking in young adulthood. Previous studies have also pointed to the importance of un-organized leisure activities, with little social control, such as attending e.g. skate parks [32] in the development of smoking habits. Nondaily smoking might be seen as a way to distinguish between oneself and “the conventional other”, which may be important in the process of constructing an identity [20]. Smoking has previously been shown to be a social marker of style [33], and though it has been increasingly stigmatized [34], recent studies suggest that smoking may still contribute to identity formation [35]. One reason may be that smoking seems to be a social behavior that may rapidly transform social action into self-identity [8,20]. Thus, the peer group, may represent a way to construct a “cool” identity, particularly when its members meet in environments that do not have smoking restrictions.

There were small initial differences between subsequent nonsmokers and nondaily smokers; the initial differences between subsequent nondaily smokers and daily smokers were more significant. Exposure to parental alcohol intoxication, weak school grades and low education predicted daily smoking rather than nondaily smoking in young adulthood, consistent with previous findings [8,10,12]. Nondaily smokers have also been shown to have higher education levels [8] and to perform better at school than daily smokers [10,12]. In our study, these factors also predicted nondaily smoking in young adulthood but not daily smoking, indicating that the two are qualitatively different phenomena.

Previous studies suggest that nondaily smokers often define themselves as nonsmokers not addicted to nicotine, and claim they can easily quit smoking [6]. One would therefore expect nondaily smokers to resemble non-nicotine-dependent daily smokers. However, in our longitudinal analyses, the variables that differentiated between nondaily smokers and nicotine-dependent daily smokers in young adulthood were the same as those that differentiated between nondaily and nondependent daily smokers. This could indicate that the key dividing characteristic uncovered in our data is whether smoking is a nondaily or daily behavior, rather than the degree of nicotine dependence. Daily smoking may, irrespective of dependence, represent a lack of control and a weakness. Thus, nondaily smoking in young adulthood may paradoxically indicate a high degree of control, with individuals able to use a highly addictive substance without becoming addicted [20].

Previous smoking status predicted subsequent nondaily smoking, and the predictive capacity of many of the family and individual factors disappeared or were attenuated when we adjusted for previous smoking. Adjustment for previous smoking provides more accurate information about the temporal relationship between potential predictors and subsequent smoking behavior. Because few predictors remained significant after adjustment, the present study cannot determine whether several presumed risk factors precede smoking behavior, or whether they are concomitants.

### Limitations

The study was population based with a longitudinal design and high response rate. However, several limitations are present. First, 40% of the sample was lost to follow-up, and some selection bias may have occurred. Second, the statistical power of some multinomial analyses may have been low as some groups had somewhat small sample sizes. Third, because of relatively long time spans between the waves, we did not obtain information about short-term stability. Some subjects might have changed smoking status between the measurements with no opportunity for us to identify these changes. Fourth, the questionnaire used did not assess more detailed information about smoking, and it was not possible to identify the smoking patterns of nondaily smokers in more detail. Finally, the findings of the present study might not be fully generalizable to the current situation in Norway or other countries as the follow-up ended in 2005, when the tobacco control context and the smoking patterns were somewhat different from the present. However, population-based findings from Norway show that the prevalence of nondaily smoking has been stable at about 10% for several decades. Also, our finding that nondaily smokers resemble non-smokers more than they resemble daily smokers, are consistent with international research. Thus, it seems reasonable that our findings also may shed some light on nondaily smokers in other countries and in more recent years.

## Conclusion

The stability of nondaily smoking during young adulthood was lower than observed in previous studies. Young adult nondaily smokers were also quite similar to nonsmokers with regard to individual and family factors. Nondaily smokers were, however, clearly different from daily smokers with regard to typical risk factors for smoking, such as low parental socioeconomic status, parental smoking and participants’ own educational achievement. Taken together, the findings suggest that nondaily smoking in young adulthood may often be temporarily, influenced by earlier smoking habits and other factors, like peer role models and parental factors.

## Competing interests

The authors declare that they have no competing interests.

## Authors’ contributions

EK, WP and TvS have all contributed to the conception and design of the study and analysis and interpretation of data. EK was responsible for the data analyses, and EK has drafted the article and WP and TvS have revised it critically for important intellectual content. All authors have read and approved of the submitted manuscript.

## Pre-publication history

The pre-publication history for this paper can be accessed here:

http://www.biomedcentral.com/1471-2458/14/123/prepub
